# The Role of Intestinal Barrier Function in Overweight Patients with IBS with Diarrhea Undergoing a Long-Term Low Fermentable Oligo-, Di-, and Monosaccharide and Polyol Diet

**DOI:** 10.3390/nu15214683

**Published:** 2023-11-04

**Authors:** Michele Linsalata, Giuseppe Riezzo, Antonella Orlando, Benedetta D’Attoma, Laura Prospero, Antonia Ignazzi, Giuseppe Losurdo, Alfredo Di Leo, Gianluigi Giannelli, Francesco Russo

**Affiliations:** 1 Functional Gastrointestinal Disorders Research Group, National Institute of Gastroenterology IRCCS “S. de Bellis”, 70013 Metropolitan, Italy; michele.linsalata@irccsdebellis.it (M.L.); giuseppe.riezzo@irccsdebellis.it (G.R.); antonella.orlando@irccsdebellis.it (A.O.); benedetta.dattoma@irccsdebellis.it (B.D.); laura.prospero@irccsdebellis.it (L.P.); antonia.ignazzi@irccsdebellis.it (A.I.); 2 Section of Gastroenterology, Department of Precision and Regenerative Medicine and Ionian Area, University of Bari, 70124 Bari, Italy; giuseppelos@alice.it (G.L.); alfredo.dileo@uniba.it (A.D.L.); 3 Scientific Direction, National Institute of Gastroenterology IRCCS “S. de Bellis”, 70013 Metropolitan, Italy; gianluigi.giannelli@irccsdebellis.it

**Keywords:** intestinal barrier, irritable bowel syndrome, low FODMAP diet, overweight, gastrointestinal symptoms

## Abstract

Overweight and obesity have been suggested as significant factors in irritable bowel syndrome (IBS) development. However, the relationship between overweight/obesity and IBS is unclear. It is known that a modified intestinal barrier, especially the permeability of the small intestine (s-IP), can play a significant role in the pathogenesis of both obesity and IBS. Moreover, dietary interventions are essential for treating both pathologies. We evaluated the gastrointestinal (GI) symptoms and the urinary and circulating markers of GI barrier function and integrity, the markers of intestinal dysbiosis and bacterial translocation, in 40 IBS patients with predominant diarrhea (IBS-D) (32 females and 8 males; mean age = 43.5 ± 1.4 years), categorized using their Body Mass Index levels as normal (NW) and overweight (OW). Evaluations were performed before and after 12 weeks of a Low FODMAP Diet (LFD). At the baseline, OW patients showed a significantly higher s-IP than NW. After an LFD, a significant improvement of s-IP in OW patients occurred, along with a significant decrease in markers of epithelial integrity and bacterial translocation. Our findings highlight the close relationship between overweight and the intestinal barrier and support their involvement in IBS-D pathophysiology. Furthermore, the positive role of an LFD in managing overweight IBS-D was highlighted.

## 1. Introduction

Irritable bowel syndrome (IBS) is a functional gastrointestinal disorder characterized by abdominal pain and changes in bowel habits without organic causes. The pathophysiology of IBS is partly understood, with associated factors including lifestyle, mental illness, and chronic inflammation [1]. Overweight and obesity have also been suggested as significant factors in IBS development [2]. Epidemiological data show a higher prevalence of IBS symptoms, such as abdominal discomfort or pain and altered bowel habits, among obese individuals compared with those with normal weight [3]. The link between obesity and IBS may be influenced by various factors, including nutrition, chronic inflammation, psychological factors, gastrointestinal (GI) hormones, and gut microbiota [4]. The current state of knowledge does not conclusively establish the causal relationship between obesity as a predisposing factor for IBS or whether IBS itself may impart an increased susceptibility to obesity.

The debate remains open on this issue, as it is hypothesized on the one hand that it may simply be two concomitant pathologies, or that there is a real association, as widely demonstrated in the case of gastroesophageal reflux, pancreatitis, chronic liver diseases, and inflammatory bowel diseases [5].

A high prevalence of IBS, approximately three times higher than that in the general population [6], is observed in tertiary care obesity centers. Obese subjects are 2.6 times more likely to have IBS than normal-weight patients [7], and women have a 1.5–3 times higher incidence of IBS than men [8]. However, not all clinical studies in patients with general or abdominal obesity have demonstrated a clear association with IBS [5]. Therefore, these data suggest the association between IBS and obesity, but the role of obesity in modulating the symptom profile of IBS is not yet clear [9]. Consequently, it is unknown whether a different diagnostic and therapeutic approach should be considered in obese patients with IBS compared with normal-weight patients.

Furthermore, the precise clinical profile and characteristics characterizing individuals with concomitant IBS and overweight remain undetermined. This aspect is particularly relevant, given that being overweight frequently precedes a more severe disease such as obesity.

Recently, research has indicated that intestinal permeability is linked to body weight, and accumulating evidence from animal models and human studies supports the involvement of intestinal barrier alterations in obesity and metabolic syndrome as well as IBS [10]. Altered gut permeability can allow the passage of luminal contents into the underlying tissues and, subsequently, into the bloodstream, leading to gut inflammation and the activation of the immune response [11].

Several reports have provided compelling evidence of a compromised intestinal barrier associated with low-grade inflammation in the upper intestinal mucosa among patients with IBS [12]. Notably, data from the literature indicate that in the IBS with diarrhea (IBS-D) variant, which accounts for approximately one-third of all IBS cases, small intestinal permeability (s-IP) is more pronounced and is linked to visceral hypersensitivity, suggesting a breakdown of the epithelial barrier as an early event [13].

Nonetheless, intestinal barrier injury and low-grade inflammation seem to represent a feature not always detectable in IBS-D patients. In our previous study, approximately half of IBS-D patients exhibited increased s-IP despite the absence of significant differences in the symptom profile, suggesting that two distinct IBS-D subtypes could be identified based on intestinal barrier impairment [14].

Recently, clinical research has shown that weight reduction in obese and overweight patients improves impaired intestinal permeability and notable enhancements in the α-diversity of the gut microbiota [15,16]. Interestingly, all these data raise the possibility that an impaired intestinal barrier, especially s-IP, could be the link between obesity and some aspects of IBS, such as symptoms profile and bowel transit [17]. However, there are no data regarding the barrier condition in IBS patients with overweight, which precedes obesity.

The oral lactulose/mannitol (Lac/Man) permeability test is a commonly used assay to investigate s-IP. This non-invasive test measures the ability of lactulose (Lac) and mannitol (Man), two sugar molecules, to pass non-metabolized through the intestinal mucosa. Lac, a di-saccharide, is absorbed through cell junctions and provides information on the paracellular pathway, while Man, a monosaccharide, is primarily absorbed across epithelial cell membranes and reflects the transcellular route. Both sugars are then excreted in the urine, and chromatography is used to determine their excretion levels.

In clinical practice, an elevated lactulose-to-mannitol ratio indicates dysfunction in s-IP. Sucrose (Suc) is another sugar probe used to evaluate intestinal barrier function, and its absorption and excretion levels are related to gastroduodenal permeation [18].

Zonulin, a human protein, plays a crucial role in regulating s-IP by inducing the opening of tight junctions (TJs). Therefore, serum and fecal zonulin levels are considered useful markers of intestinal barrier function [19].

Additionally, two potential markers of intestinal barrier integrity are circulating levels of intestinal fatty acid binding protein (I-FABP) and diamine oxidase (DAO), as these proteins are synthesized in enterocytes [20].

The association between obesity and IBS may also involve nutritional factors [21]. Dietary interventions are essential for treating IBS and obesity, and certain foods have been identified as potential triggers for IBS in obese individuals [22]. Research suggests that reducing fermentable oligo-, di-, and monosaccharides and polyols (FODMAPs) in the diet can alleviate IBS symptoms and improve intestinal barrier function [23]. Yet, there are a lack of data on the effects of a low FODMAP diet (LFD) in overweight IBS patients.

This study investigated urinary and circulating markers of GI barrier function in patients with IBS-D, comparing normal-weight (NW) and overweight (OW) patients. Two main objectives were addressed: first, to understand if changes in GI barrier function are related to overweight status, and second, to determine if an LFD can differently modify intestinal barrier characteristics in NW and OW IBS patients.

The urinary marker of intestinal dysbiosis, indican, and the circulating marker of bacterial translocation, lipopolysaccharide (LPS), were also evaluated.

## 2. Materials and Methods

### 2.1. Patient Recruitment

IBS-D patients, diagnosed according to the Rome IV criteria, were recruited in this prospective study between January 2021 and September 2022 from the outpatients of the Functional Gastrointestinal Disorder Unit—National Institute of Gastroenterology “S. de Bellis” Research Hospital in Apulia, a Mediterranean region in south-eastern Italy.

Patients underwent a physical examination, completed the “Gastrointestinal Symptom Rating Scale” (GSRS) questionnaire [24], and provided basal urinary and blood samples for various tests. Eligibility was determined through gastroscopy, colonoscopy, fecal occult blood tests, stool culture, and stool ova and parasite tests. Female patients provided samples during the follicular phase of their menstrual cycle to prevent interference and contamination.

The inclusion criteria were an age 18 years or older, Body Mass Index (BMI) between 18.5 and 29.9 kg/m^2^, symptoms resembling IBS-D for at least 12 weeks, a specific stool pattern according to Schmulsson [25], a minimum average score of >75 on the IBS-Severity Scoring System (IBS-SSS), and no dietary restrictions. Patients positive for anti-endomysium and tissue transglutaminase antibodies were excluded to avoid potential non-celiac gluten-sensitivity cases. Patients with HLA-DQ2/HLA-DQ8 negative/negative were included to prevent the occurrence of symptoms due to non-celiac gluten-sensitivity (NCGS) observed in some IBS patients with the presence of HLA-DQ2 and -DQ8 [26].

Exclusion criteria comprised pregnancy, intense physical activity, constipation, fever, post-infectious IBS, giardiasis, previous abdominal surgery, endocrine and metabolic disorders, cardiovascular diseases, altered hepatic and renal functions, intestinal atrophy due to secondary causes, previous neoplasm diagnosis, use of drugs for IBS symptom relief or probiotics two weeks before evaluation, prior antibiotic therapy, and use of selective serotonin reuptake inhibitors or other antidepressants. Patients with a history of IBD were excluded.

All subjects provided written informed consent for data collection, and the reasons for research interruptions were recorded. The clinical trial was registered on http://www.clinicaltrials.gov (NCT03423069); the last date of data access was 22 March 2022. The study was a component of a research project approved by the local Scientific Committee and the Institutional Ethics Committee of IRCCS Ospedale Oncologico—Istituto Tumori Giovanni Paolo II, Bari, Italy (N. 274/C.E. 12.12.17).

### 2.2. Study Design

The study design included three visits as already described in our previous manuscript [23]. During the first visit (Baseline), participants underwent a gastroenterological exam and provided informed consent after learning about the study’s objectives, which focused on the effectiveness of a 12-week diet plan for reducing IBS symptoms. Qualified nutritionists assessed the subjects’ food habits, lifestyle, and health issues. The participants were asked to maintain their regular diets while keeping a daily food journal for seven days. This included recording stool characteristics [27], medication use, physical activity, and food intake to estimate energy consumption.

At visit 2 (attribution to diet), anthropometric measures were taken a week after the first visit. Participants completed the IBS-SSS questionnaire [28], and those with a total score greater than 75 were enrolled in the trial. The daily food diary from the previous week was reviewed to reassess inclusion and exclusion criteria. Personalized diets were provided by qualified nutritionists, and participants were encouraged to maintain daily journals throughout the diet, tracking various factors, including diet, sexual behavior, exercise, medications, and stool characteristics. Each patient provided stool, urine, and blood samples for analytical measurements and the sugar absorption test (SAT).

At visit 3 (final visit), researchers collected symptoms and food questionnaires completed during the 12 weeks of the diet. Patients were given the IBS-SSS and IBS diet-adherence report scale—food diary (IDARS) to monitor diet compliance. All the patients underwent the same procedures as at visit 2 for the anthropometric and biochemical measurements.

### 2.3. Symptom Profile

The symptom profile was evaluated using the validated GI symptoms questionnaire, IBS-SSS [24]. Intestinal habits were recorded according to the Bristol stool form chart [29].

This questionnaire comprehensively evaluates the severity of IBS symptoms by rating five items on a visual analog scale. The five items were “Abdominal pain severity”, “Abdominal pain frequency”, “Abdominal distension severity”, “Dissatisfaction with bowel habits”, and “Impact of symptoms on quality of life”. Each symptom was given a rating out of 100. Patients chose a point on the line representing their feelings for items 1 through 4, and the distance from zero was used to calculate the score (which ranged from 0 to 100). The last item (5) asked for the percentage of days out of ten on which the individuals reported experiencing “Abdominal pain”.

To produce a metric scale from 0 to 100, the answer was multiplied by ten. The aggregate of the five items made a final score that ranged from 0 to 500. According to scores, cases were classified as “mild” (75 to 175), “moderate” (175 to 300), and “severe” (>300). Conventionally, healthy subjects have scores below 75, and patients should be regarded as being in a remission phase if their scores are below 75.

### 2.4. Assessment of Nutrient Intake

To assess their calorie intake and expenditure, the patients had to keep a food diary both before the trial and throughout the diet intervention. The diary included the amounts (reported in grams) and types of food taken daily at breakfast, lunch, dinner, and snacks, as well as the nature and length of physical activity [30]. Food diaries kept both before and during the program were examined by nutritionists. Utilizing specialized software (Progetto Dieta v. 2.0, available at http://www.progettodieta.com data last viewed on 18 March 2020), the weight and proportion of daily carbs, lipids, proteins, and dietary fiber, as well as the percentage of alcohol consumption and the daily energy intake and consumption expressed in kcal, were calculated.

### 2.5. Assessment of the Anthropometric Profile

The following anthropometric parameters were evaluated: height, weight, BMI, and abdominal and waist circumferences. An SECA mod. 700 mechanical column scale and an SECA mod. 220 altimeter (INTERMED S.r.l., Milan, Italy) were used to assess the subjects’ weight and height to calculate their BMI (kg/m^2^). A mod—201 SECA tape measure was used to calculate the waist and abdominal circumferences. All patients who underwent Body Impedance Assessment (BIA) had fasted for at least 4 h and abstained from alcohol and strenuous exercise for 12 h [31]. Body cell mass (BCM), fat-free mass (FFM), fat mass (FM), total body water (TBW), and extracellular water (ECW) were calculated from Rz and Xc using specialized software (Bodygram PLUS Software v. 1.0, Akern SRL, Pontassieve, FI, Italy). Phase angle (PhA), calculated as the arctangent of the Xc/Rz ratio, was also calculated.

### 2.6. Intervention Diet

Following a review of the food diaries and in-person individual counseling with the nutritionists at visit 2, a customized LFD was assigned. A restricted intake of FODMAPs is implied by LFD [32]. Utilizing specialized software (Nutrigeo 8.6.0.0, Progeo Medical, Centobuchi di Monteprandone, AP, Italy), the daily intake of macronutrients (20% proteins, 30% fats, and 50% glucides) was assessed. The diets were created as described elsewhere [30]. Each patient received a comprehensive weekly menu consisting of breakfast, lunch, and dinner in addition to two light snacks in the afternoon and mid-morning. The menu was accompanied by a brochure that included detailed information on foods that were allowed and prohibited as well as which ones should be reduced based on recommendations from Monash University [33]. Alcohol use was discouraged, and a sufficient intake of fiber was assured.

### 2.7. Sugar Absorption Test (SAT)

All participants in the research underwent an s-IP assessment using an SAT after fasting overnight. Before the test, urine samples were obtained in our laboratory to examine the potential presence of natural sugars. Next, participants consumed a solution containing 10 g of Lac, 5 g of Man, and 40 g of Suc in 100 mL of liquid. Urine samples were collected up to 5 h after the solution intake.

Individual urine volumes were measured and recorded. A portion of 2 mL was taken from each sample, thoroughly mixed, and stored at −80 °C until analysis. Chromatographic analysis was carried out to determine the levels of the three sugar probes (Lac, Man, and Suc) in the urine, following the method previously described by our research group [23]. The percentages of ingested Lac (%Lac), Man (%Man), and Suc (%Suc) were assessed, and for each sample the Lac/Man ratio was calculated. Values above 0.03 were considered impaired [34].

### 2.8. Biomarkers of Intestinal Barrier Function and Integrity

Biochemical assessments were carried out at the beginning and conclusion of the dietary intervention. Serum samples and crude stool samples from patients in the study were frozen and stored at −80 °C within 12 h of collection.

Serum and fecal zonulin levels were determined using ELISA kits from Immunodiagnostik AG (Bensheim, Germany). The manufacturer’s guidelines were followed, and values below 48 ng/mL for serum and 107 ng/mL for fecal samples were considered normal. DAO and I-FABP serum concentrations were assessed using ELISA kits from Cloud-Clone Corp. (Houston, TX, USA) and Thermo Fisher Scientific (Waltham, MA, USA), respectively.

### 2.9. Biomarkers of Intestinal Dysbiosis and Bacterial Translocation

Urinary indican levels were assessed with a standard colorimetric assay kit (indican assay kit, ABNOVA Corporation, Taipei, Taiwan). Indican levels above 20 mg/L were considered indicative of fermentative dysbiosis [35]. To measure serum LPS, an ELISA kit from Cloud-Clone Corp. (Katy, TX, USA) was employed.

### 2.10. Statistical Analysis

Unless stated otherwise, all results are presented as means ± SEM. Due to the small sample size and to prevent the assumption of a normal distribution from being violated, nonparametric tests were carried out. To identify changes between the biochemical parameters and the items on the IBS-SSS questionnaire before and after the low FODMAP diet (LFD), the Wilcoxon rank-sum test was employed in either the IBS-D patients as a whole or in subgroups initially classified based on BMI (>25 or <25). The two groupings were compared both before and after the diet using the Mann–Whitney test. BMI was compared to every other variable using the Spearman rank correlation test. All the differences were considered significant at a 5% level. A specific statistical package (2005 Stata Statistical software release 9; Stata Corp., College Station, TX, USA) was used.

## 3. Results

### 3.1. Number, Anthropometric, and Nutritional Information of the Patients and Intervention Diet

**Figure 1:**
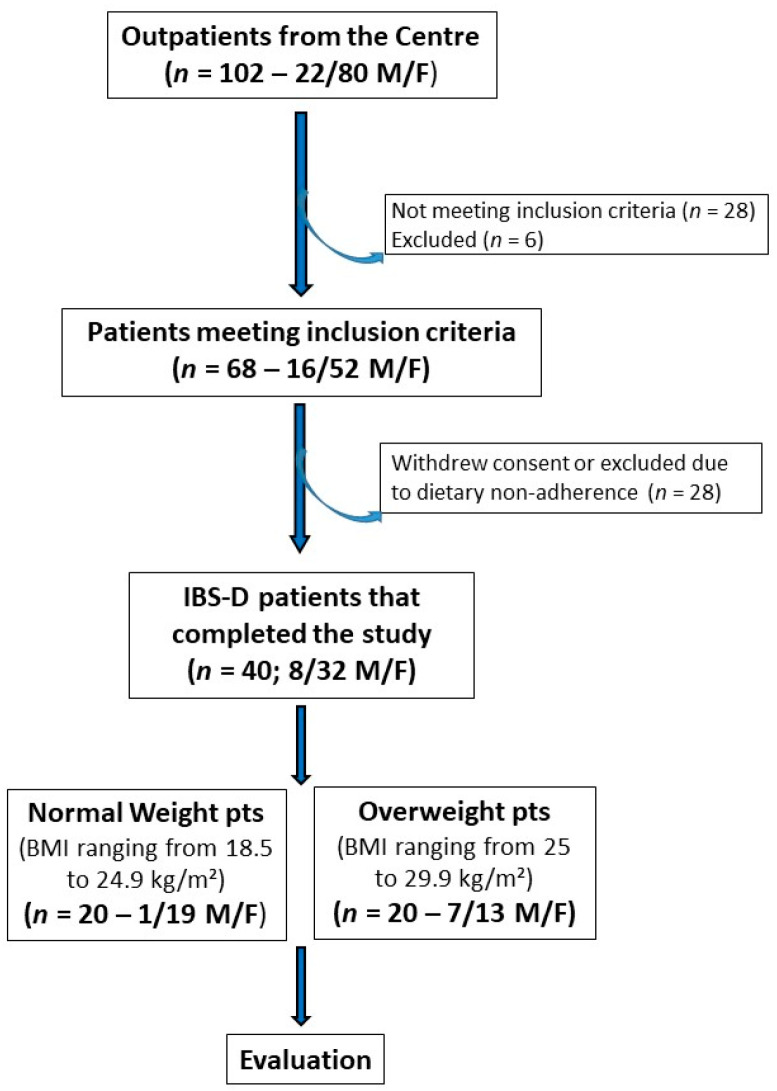
The flowchart of the study.

Figure 1 depicts the patient flow throughout the study. Initially, 102 subjects experiencing IBS-D (80 females and 22 males) were recruited, and 68 individuals (52 females and 16 males) met the inclusion criteria. The IBS cohort was then categorized based on BMI into an NW subgroup (BMI ranging from 18.5 to 24.9 kg/m²) and an OW subgroup (BMI ranging from 25 to 29.9 kg/m²). Ultimately, 40 patients (32 females and 8 males; mean age = 43.5 ± 1.4 years) completed the 12-week study following the LFD. Of these 40 patients, 20 were NW patients (19 females and 1 male; mean age = 40.9 ± 2.2 years) and 20 were OW patients (13 females and 7 males; mean age = 46.0 ± 1.65 years).

Table 1 reports the anthropometric characteristics of the patients at the beginning of the study. At baseline, the BMI significantly differed between NW and OW patients (*p* < 0.0001). In the entire group of patients with IBS-D, the BMI decreased significantly by approximately 5.5% (25.98 ± 0.76 kg/m² vs. 24.55 ± 0.75 kg/m², *p* < 0.0001) at the end of the dietary treatment compared with the initial measurements. Within the OW subgroup, there was a significant decrease, by approximately 6.05%, in BMI after the diet compared with baseline (29.57 ± 0.95 kg/m² vs. 27.78 ± 1.02 kg/m², *p* < 0.0001)), although it remained above the normal BMI range (24.9 kg/m²).

The NW subgroup also experienced a significant decrease, by approximately 4.74%, in BMI after the LFD (22.39 ± 0.38 kg/m² vs. 21.33 ± 0.43 kg/m²; *p* < 0.0001), though to a lesser extent than the OW subgroup.

Despite the significant decrease in BMI in the OW subgroup at the end of the LFD, the BMI remained significantly higher than that in the NW subgroup (27.78 ± 1.02 kg/m² vs. 21.33 ± 0.43 kg/m²; *p* < 0.0001).

No correlation was observed comparing BMI versus all other variables in the total group and the two subgroups considered.

Table 2 shows patients’ main daily nutritional information pre-LFD and post LFD, respectively. Even if the energy consumption was similar before and after the diet, a reduction in energy intake, a reduction in lipids, and an increase in proteins and carbohydrates were evident. Last but not least, a clear decrease in the intake of dietary fibers was present.

### 3.2. The Symptom Profile in IBS-D Patients

The LFD diet had a notable impact on all individual item scores and the total score on the IBS-SSS questionnaire in the whole group of IBS-D patients. Specifically, the IBS-SSS total score decreased by 46.21% after 12 weeks of treatment (270.50 ± 14.10 and 145.50 ± 16.06, before and after diet; *p* < 0.0001).

When evaluating the IBS-SSS parameters based on BMI subgroups (Table 3), it was found that NW and OW patients did not exhibit significantly different symptom profiles at the beginning of the study. However, the diet significantly altered the IBS-SSS scores in both subgroups of patients. Specifically, the total IBS-SSS score was reduced by 47.25% in the NW subgroup and by 45.01% in the OW subgroup. At the end of the study, there were no significant differences in the symptom profiles expressed as IBS-SSS parameters between NW and OW patients.

### 3.3. The Small Intestinal Permeability (s-IP)

The s-IP in IBS-D patients was evaluated using SAT before and after treatment. In the entire IBS-D group, the %Lac values significantly decreased after treatment (0.32 ± 0.04 vs. 0.22 ± 0.02; *p* < 0.0001). Similarly, the %Man rate significantly reduced at the end of treatment (14.05 ± 0.51 vs. 12.60 ± 0.51; *p* = 0.008). Consequently, the Lac/Man ratio decreased significantly by 22.7% after the diet (0.023 ± 0.002 vs. 0.017 ± 0.001; *p* = 0.003). The %Suc also reduced in a significant manner at the end of the diet (0.23 ± 0.05 vs. 0.18 ± 0.04; *p* = 0.016).

**Figure 2:**
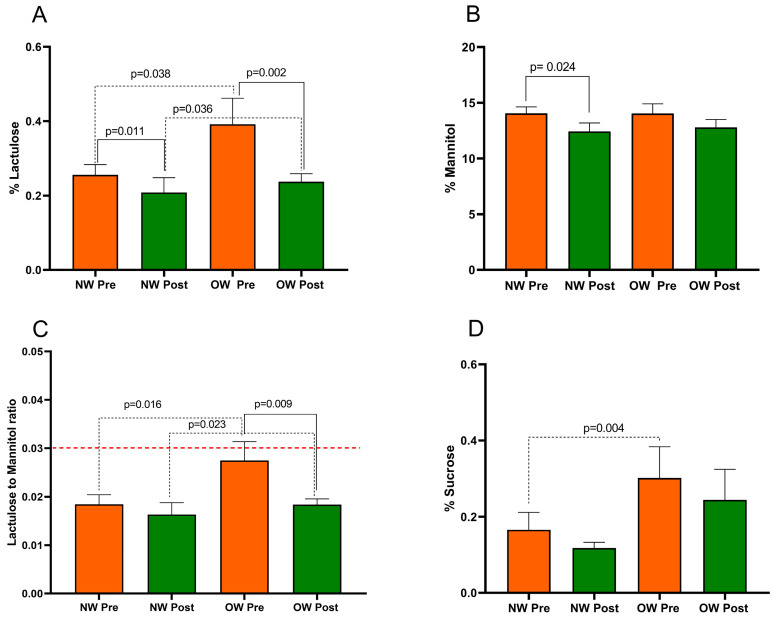
The intestinal permeability parameters in IBS-D patients categorized into normal weight (NW) and overweight (OW) subgroups according to their BMI at baseline, before (pre) and after (post) 12 weeks of a low FODMAP diet (LFD). Panel (**A**) =% lactulose; panel (**B**) =% mannitol; panel (**C**) = lactulose to mannitol ratio; panel (**D**) =% sucrose. The data are expressed as Mean ± SEM. The Wilcoxon signed-rank test was used to compare the pre-treatment and post-treatment data. The Mann–Whitney test was applied to compare the two subgroups before and at the end of the diet. Differences are considered significant at *p* < 0.05. The red dotted line indicates the cut-off value for s-IP (<0.030).

Figure 2 presents the SAT parameters in IBS-D patients categorized according to BMI into NW and OW subgroups. The Mann–Whitney test revealed a significant difference in %Lac, the Lac/Man ratio, and %Suc between the NW and OW subgroups at baseline (*p* = 0.038, *p* = 0.016, and *p* = 0.004, respectively). Notably, at the start of the study, the OW subgroup had a mean Lac/Man ratio of 0.027, a value close to the cutoff value of 0.030 for pathological s-IP. No difference in %Man (*p* = 0.732) was found between the sub-groups at baseline.

After the LFD, in OW patients, the %Lac was reduced by 39% (*p* = 0.002). However, %Man did not show a significant difference (*p* = 0.128). Consequently, the Lac/Man ratio in OW patients significantly decreased by 33% (*p* = 0.009), reaching a value similar to that in the NW ones. Regarding the NW group, there was a significant reduction in %Lac and %Man (*p* = 0.011 and *p* = 0.024, respectively) without a significant reduction in the Lac/Man ratio in the NW group after the diet (*p* = 0.141).

Finally, the %Suc was reduced by the LFD in both OW patients (*p* = 0.061) and NW ones (*p* = 0.319) without reaching statistical significance.

At the end of the study, the SAT parameters did show differences in the %Lac and Lac/Man ratio between the two subgroups (*p* = 0.036 and *p* = 0.023, respectively). No differences in %Man (*p* = 0.752) and %Suc (*p* = 0.149) were found between the subgroups.

### 3.4. Biomarkers of Intestinal Barrier Function and Integrity

In the whole IBS-D group, serum zonulin and I-FABP were quite similar before and after the diet (29.93 ± 0.77 ng/mL vs. 28.57 ± 0.88 ng/mL; *p* = 0.133 and 2.86 ± 0.62 ng/mL vs. 2.58 ± 0.50 ng/mL; *p* = 0.649, respectively). On the contrary, fecal zonulin decreased significantly after the diet (168.10 ± 12.38 ng/mL vs. 139.80 ± 9.52 ng/mL; *p* = 0.018) as well as DAO (38.47 ± 1.26 ng/mL vs. 36.83 ± 1.26 ng/mL; *p* = 0.015).

**Figure 3:**
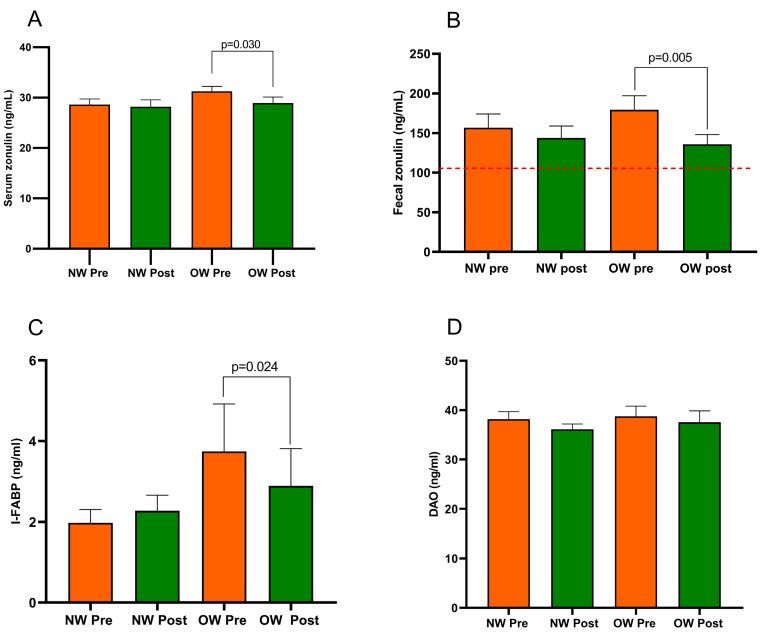
Biomarker of the intestinal barrier function and integrity in IBS-D patients categorized into normal weight (NW) and overweight (OW) subgroups according to their BMI at baseline, before (pre) and after (post) 12 weeks of a low FODMAP diet (LFD). Panel (**A**) = serum zonulin; panel (**B**) = fecal zonulin; panel (**C**) = Intestinal-Fatty Acid Binding Protein, I-FABP; panel (**D**) = Diamine Oxidase, DAO. Data are expressed as Mean ± SEM. Wilcoxon signed-rank test was used to compare pre-treatment and post-treatment data. The Mann–Whitney test was applied when comparing the two subgroups before and at the end of the diet. Differences are considered significant at *p* < 0.05. The red dotted line indicates the cut-off value of fecal zonulin (107 ng/mL).

Figure 3 describes the profile of the markers of intestinal barrier function and integrity (fecal and serum zonulin, I-FABP, and DAO) recorded in the IBS subgroup. The comparison of the two subgroups at baseline showed no differences in all the biomarkers. Regarding the diet’s effect, the NW subgroup did not show any changes. At the same time, the OW group of IBS-D patients had significantly lower serum and fecal zonulin levels (*p* = 0.030 and *p* = 0.005, respectively) and a reduced concentration of I-FABP (*p* = 0.024) but not DAO (*p* = 0.055). Before and after the diet, the fecal zonulin concentrations were significantly higher than the 107 ng/mL threshold value. There were no differences in the post-diet concentrations of any biomarkers between the subgroups.

### 3.5. Biomarkers of Intestinal Dysbiosis and Bacterial Translocation

Regardless of BMI, the indican contents in the urine of IBS-D patients were significantly higher than the 20 mg/mL limit both before and after treatment. Nonetheless, at the conclusion of the LFD, there was a noteworthy drop in the indican urine concentration across all IBS-D patients (70.43 ± 6.71 versus 59.32 ± 5.23, *p* = 0.046). As regards the effect of diet, both the NW and the OW groups showed no statistically significant changes (68.95 ± 8.89 vs. 60.34 ± 7.43, *p* = 0.226; 71.90 ± 10.27 vs. 58.30 ± 7.53, *p* = 0.141, respectively). No difference in the indican urinary levels was found between the NW and OW subgroups at baseline and the end of the diet.

Finally, within the entire cohort of IBS-D patients, the beginning and end of the study LPS concentrations differed substantially (0.057 ± 0.01 ng/mL vs. 0.051 ± 0.010 ng/mL; *p* = 0.0005).

**Figure 4:**
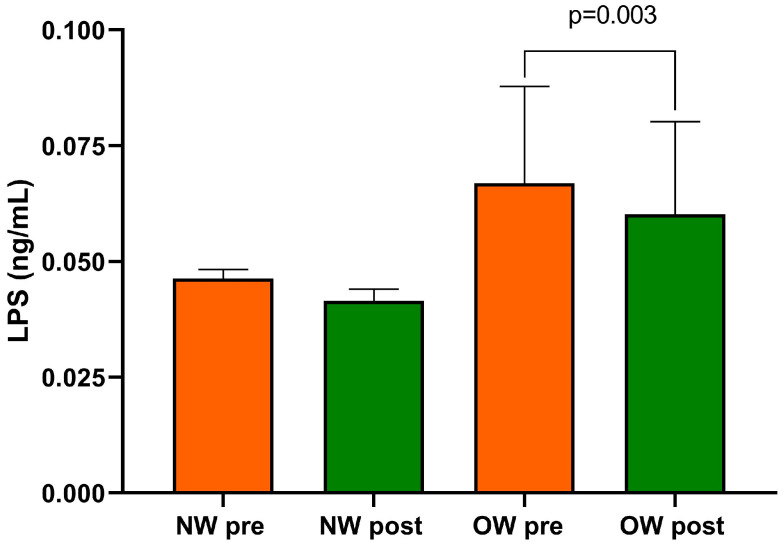
Serum LPS level in IBS-D patients categorized into normal weight (NW) and overweight (OW) subgroups according to their BMI at baseline, before (pre) and after (post) 12 weeks of a low FODMAP diet (LFD). Data are expressed as Mean ± SEM. Wilcoxon signed-rank test was used to compare pre-treatment and post-treatment data. The Mann–Whitney test was applied when comparing the two subgroups before and at the end of the diet. Differences are considered significant at *p* < 0.05.

After categorizing for BMI levels, baseline LPS was higher in the OW subgroup compared with the NW one without reaching statistical significance. As an effect of diet, only the OW subgroup showed significantly reduced levels of LPS compared with baseline (*p* = 0.003) (Figure 4). At the end of the diet, no statistically significant difference in LPS between the two subgroups was found.

## 4. Discussion

It is now known that excess body weight not only negatively impacts the progression of multiple diseases, such as cardiovascular diseases, diabetes, autoimmune disorders, and neoplasms, but also affects gut diseases, including colorectal cancer and IBD [36]. Nevertheless, few studies have examined the association between overweight/obesity and IBS, and it has not been determined whether obesity precedes IBS or vice versa. Given the substantial medical implications of obesity and recognizing that the precursor of this condition, namely overweight, is not consistently considered, we directed our attention to this critical focus in the present research.

The findings from this study underscore the strong connection between alterations in GI barrier function observed in patients with IBS-D and excessive body weight. Additionally, it highlights the potential benefits of the LFD in effectively managing this specific subset of IBS-D patients.

At the start of the study, the entire group of IBS-D patients was classified according to the World Health Organization’s guidelines. Those with a BMI equal to or higher than 25 kg/m² were categorized as OW, while those with a lower BMI were classified as NW [37].

Evaluating the IBS-SSS questionnaire, the total symptom and single-item scores did not show significant differences between the OW and NW groups. These results differed from those by Sadik et al. [17], who claimed more intense GI symptoms in patients with a higher BMI. These discrepancies may depend on the type of patient involved, overweight versus obese, the intensity of the symptoms, and the questionnaire used. However, the lack of a significant difference in the symptom profile did not exclude an s-IP impairment, as already reported in our previous paper on IBS [14]. Indeed, among OW patients, the baseline s-IP, expressed as Lac% and Lac/Man ratio, exhibited higher values than those in NW individuals. Additionally, the %Suc was notably elevated in OW patients compared with their NW counterparts. These results corroborate the strong association between excess body weight and the intestinal barrier that has been shown in prior in vitro and in vivo research [38]. However, our results did not show evidence of “leaky gut” syndrome, probably because only OW patients were involved, but a more pronounced alteration in the paracellular pathway in the OW patients was evident. In line with these data, a recent study observed an alteration in GI permeability and increased paracellular permeability in obese mice [39]. These data suggest that the pathophysiologic mechanism underlying IBS-D may differ, particularly regarding the interplay between weight and intestinal permeability.

In addition to the observed changes in s-IP, another pivotal finding is the presence of intestinal dysbiosis, which appears to be a shared characteristic among IBS and obese patients [40]. Our study revealed that the concentration of indican in the urine of individuals with IBS-D exceeded the threshold of 20 mg/mL at baseline, regardless of their BMI. This finding confirms the presence of fermentative dysbiosis in both subgroups. In the present study, the evaluation of skatole as a marker of putrefactive dysbiosis was not performed due to the constant normal values found in IBS-D patients in our previous studies [14,23]. Notably, LPS, an indicator of bacterial translocation, exhibited slightly higher levels in OW individuals than NW ones, although this difference was not statistically significant. This suggests an initial disturbance in the microbiota of OW patients, which could become more pronounced in individuals with obesity [41].

Regarding how the LFD affects IBS, a number of studies in the literature suggest that consuming fewer FODMAPs may help with symptoms and intestinal barrier function [42]. The whole IBS-D group in our study experienced a considerable reduction in symptoms after 12 weeks of the LFD, which is consistent with other research [23,30,43] about its effectiveness.

Following the LFD, there were discernible drops in weight, BMI, and the circumferences of the waist and abdomen. These decreases were probably brought on by the diet’s length and limitations. A long-term, customized diet may result in weight loss and a lower BMI, especially in relation to the so-called “elimination phase” [30].

The improvement in the symptom profile may be attributed to the diet per se and/or improved intestinal barrier health conditions. Remarkably, both s-IP and intestinal barrier integrity exhibited substantial enhancement upon completing the dietary intervention. These results, along with a noteworthy decrease in the indicators of dysbiosis and bacterial translocation, suggest that the LFD may be beneficial in the management of IBS-D and reinforce the significance of these variables in the pathophysiology of the disease.

There are no available data regarding the effect of the LFD on excess body weight in IBS patients. When we categorized our patients according to BMI, the diet led to a significant decrease in this parameter in both NW and OW subgroups, although in the OW group, this value remained above 25 kg/m^2^. The diet exerted similar symptom reductions in both subgroups, but there were clear differences regarding s-IP parameters and markers of mucosal integrity. Although the two groups started from an s-IP below the cut-off for impaired permeability, our data show that OW and NW subgroups significantly reduced Lac%, while Man% decreased only in the NW group. Consequently, Lac/Man decreased significantly only in the OW group. Thus, the LFD appears to have varying effectiveness in modifying s-IP in IBS patients, depending on their BMI levels. These data are also supported by the significant decrease in fecal and serum zonulin levels and a reduction in circulating levels of I-FABP only in the OW group. Increased circulating levels of I-FABP reflect enterocyte loss and are inversely proportional to villous atrophy. It has been reported to be an appropriate marker of intestinal permeability for obesity, characterized by rapid enterocyte turnover and shortened intestinal villi [44].

The improvement in the function and integrity of the intestinal epithelium was accompanied by a conceivable decreased translocation of Gram-negative bacteria across the intestinal villi, as suggested by the diminished circulating levels of LPS in the OW subgroup following the dietary intervention.

All these findings confirm that the highest s-IP is linked to overweight. Furthermore, the LFD effectively enhances the intestinal barrier’s function and integrity in OW patients while reducing BMI. This is likely to prevent the progression to a full-fledged condition like obesity.

The relationship between overweight and IBS is complex. Both conditions are linked to changes in gut microbiota, inflammation, and visceral sensitivity. Adipose tissue in overweight individuals produces hormones and neurotransmitters affecting gut function. Dietary habits and psychological factors also play a role [3]. It is essential to emphasize that the relationship between overweight and IBS is likely bidirectional, implying that one condition can influence the other and vice versa. For instance, IBS symptoms, especially in cases of the diarrhea-predominant variant, can prompt dietary modifications, impacting weight and contributing to weight fluctuations [45].

Nevertheless, the present research has some limitations. The patient cohort was too small to draw firm conclusions. Therefore, further research is needed to investigate the still-veiled relationship between BMI and the intestinal barrier in IBS-D patients. Moreover, the fermentative dysbiosis found in the small intestine of IBS-D patients should be supported by an appropriate analysis of bacterial populations in the GI tract, such as molecular analysis of 16S rRNA genes.

## 5. Conclusions

OW individuals with IBS-D appear to exhibit compromised intestinal barrier function. The LFD is particularly effective in restoring this impaired barrier function in this specific group of patients. Addressing weight management and IBS symptoms through tailored nutritional interventions, possibly coupled with strategies like regular physical activity, can improve the overall well-being of individuals with both conditions.

## Figures and Tables

**Table 1:** Descriptive statistics of the anthropometric characteristics of the IBS-D subjects at V1 (baseline) and V3 (final study visit).

	Total Group (*n* = 40)			Normal Weight (*n* = 20)			Overweight (*n* = 20)			
	Pre	Post	*p*	Pre	Post	*p*	Pre	Post	*p*	*p*
									(Post NW vs. Post OW)	
Weight (kg)	68.85 ± 2.19	65.03 ± 2.03	<0.0001	58.22 ± 1.61	55.67 ± 1.53	<0.0001	79.49 ± 2.30	74.40 ± 2.31	<0.0001	<0.0001
Height (m)	1.63 ± 0.01	1.63 ± 0.01	ns	1.61 ± 0.01	1.61± 0.01	ns	1.64 ± 0.02	1.64 ± 0.02	ns	ns
BMI (kg/m^2^)	25.98 ± 0.76	24.55 ± 0.75	<0.0001	22.39 ± 0.38	21.33 ± 0.43	<0.0001	29.57 ± 0.95	27.78 ± 1.02	<0.0001	<0.0001
Abdominal circumference (cm)	91.25 ± 1.75	88.19 ± 1.75	<0.0001	83.24 ± 1.62	80.52 ± 1.60	0.004	99.25 ± 1.80	95.86 ± 1.95	0.0002	<0.0001
Waist circumference (cm)	82.07 ± 2.07	79.04 ± 1.89	<0.0001	71.71 ± 1.41	70.05 ± 1.11	0.026	92.44 ± 2.09	88.04 ± 2.23	<0.0001	<0.0001
PhA (degrees)	6.06 ± 0.23	6.20 ± 0.15	0.027	5.63 ± 0.15	5.82 ± 0.17	0.053	6.49 ± 0.41	6.58 ± 0.22	ns	0.013
BCM (kg)	26.50 ± 1.19	26.22 ± 1.10	ns	22.29 ± 0.62	22.19 ± 0.61	ns	30.72 ± 1.90	30.26 ± 1.69	ns	<0.0001
FM (kg)	19.83 ± 1.20	17.88 ± 1.15	<0.0001	15.06 ± 1.02	13.52 ± 0.98	0.0009	24.59 ± 1.59	22.24 ± 1.56	0.0007	<0.0001
FFM (kg)	49.12 ± 1.54	47.39 ± 1.46	<0.0001	43.31 ± 0.95	42.17 ± 0.86	0.002	54.93 ± 2.30	52.62 ± 2.26	<0.0001	<0.0001
TBW (L)	35.78 ± 1.11	34.54 ± 1.05	<0.0001	31.55 ± 0.71	30.64 ± 0.64	0.003	40.02 ± 1.65	38.45 ± 1.58	0.0002	<0.0001
ECW (L)	16.33 ± 0.46	15.32 ± 0.37	<0.0001	15.04 ± 0.41	14.30 ± 0.39	0.003	17.62 ± 0.74	16.35 ± 0.55	0.0013	0.0092

BMI: Body Mass Index; PhA: Phase Angle; BCM: body cell mass; FM: fat mass; FFM: fat-free mass; TBW: total body water; ECW: extracellular water; NW: normal weight; and OW: overweight. Data are expressed as means ± SEM. *p*-value was determined with the Wilcoxon signed-rank test. The Mann–Whitney test was applied when comparing the two subgroups before and at the end of the diet. Differences were considered significant at *p* < 0.05; ns: not significant. Pre: baseline; Post: final study visit.

**Table 2:** Main daily nutritional information of IBS-D subjects before (Pre) and after (Post) 12 weeks of low FODMAP diet.

	Pre (*n* = 40)	Post (*n* = 40)	*p*
Energy consumption (kcal)	2149 ± 60.24	2085 ± 53.61	ns
Energy intake (kcal)	2159 ± 113.70	1572 ± 33.95	<0.0001
Basal metabolism (kcal)	1493 ± 32.89	1489 ± 28.88	ns
Proteins (g)	77.85 ± 1.73	85.61 ± 4.90	ns
Proteins (%)	16.11 ± 0.53	19.81 ± 0.12	<0.0001
Lipids (g)	88.46 ± 6.10	51.96 ± 1.13	<0.0001
Lipids (%)	36.37 ± 1.18	29.75 ± 0.14	<0.0001
Carbohydrates (g)	251.6 ± 13.30	251.8 ± 14.15	ns
Carbohydrates (%)	46.86 ± 1.44	50.13 ± 0.22	<0.01
Alcohol (%)	0.76 ± 0.26	0.31 ± 0.13	ns
Dietary fiber (g)	20.60 ± 1.37	14.21 ± 0.17	<0.0001

Data are expressed as means ± SEM; *p*-value was determined with Wilcoxon signed-rank test; differences were considered significant at *p* < 0.05; ns: not significant. Pre: diet attribution; Post: final study visit. Energy consumption: the sum of the basal metabolic rate, the thermic effect of food, and the energy expended in physical activity. Energy intake: the caloric or energy content provided by food and drink.

**Table 3:** The single-item and total scores on the IBS symptom severity scale (IBS-SSS) before (pre) and after (post) 12 weeks of a low FODMAP diet in the whole group of IBS-D patients and in subjects categorized according to BMI group.

	Total Group (*n* = 40)			Normal Weight (*n* = 20)			Overweight (*n* = 20)		
	Pre	Post	*p*	Pre	Post	*p*	Pre	Post	*p*
Abdominal pain intensity	46.25 ± 3.97	22.70 ± 3.53	<0.0001	49.60 ± 4.67	25.90 ± 4.62	<0.0001	42.90 ± 6.45	19.50 ± 5.37	0.0039
Abdominal pain frequency	46.00 ± 4.71	21.80 ± 4.09	<0.0001	48.50 ± 6.04	19.60 ± 4.36	<0.0002	43.50 ± 7.34	24.00 ± 7.01	0.0051
Abdominal distension	54.45 ± 3.83	27.83 ± 3.67	<0.0001	62.80 ± 3.49	30.50 ± 5.43	0.0001	46.10 ± 6.07	25.15 ± 5.01	<0.0078
Dissatisfaction with bowel habit	67.48 ± 3.56	37.60 ± 4.05	<0.0001	72.35 ± 4.54	39.75 ± 5.49	0.0004	62.60 ± 5.38	35.45 ± 6.06	0.0026
Interference in life in general	56.35 ± 3.65	35.53 ± 4.33	<0.0001	58.35 ± 5.51	38.00 ± 5.82	<0.0113	54.35 ± 4.88	33.05 ± 6.50	0.0041
Bristol stool-form scale	5.01 ± 0.12	3.90 ± 0.20	<0.0001	5.03 ± 0.14	4.26 ± 0.24	0.0006	4.99 ± 0.20	3.55 ± 0.33	<0.0001
Total score	270.50 ± 14.10	145.50 ± 16.06	<0.0001	291.60 ± 14.78	153.80 ± 22.030	<0.0001	249.50 ± 23.46	137.20 ± 23.81	0.0009

Data are expressed as Mean ± SEM. Wilcoxon signed-rank test was used to compare pre-treatment and post-treatment data. The Mann–Whitney test was applied when comparing the two subgroups before and at the end of the diet. No differences were found between IBS-SSS single items and the total score between NW and OW patients before and after the diet. All differences are considered significant at *p* < 0.05.

## Data Availability

The datasets used and/or analyzed during the current study are available from the corresponding author upon reasonable request.
